# Intracranial Arterial Calcification Relates to Long-Term Risk of Recurrent Stroke and Post-stroke Mortality

**DOI:** 10.3389/fneur.2020.559158

**Published:** 2020-10-09

**Authors:** Xiaohong Wu, Daniel Bos, Lijie Ren, Thomas Wai-hong Leung, Winnie Chiu-Wing Chu, Lawrence Ka Sing Wong, Jill Abrigo, Xiang Yan Chen

**Affiliations:** ^1^Department of Neurology, The First Affiliated Hospital of Shenzhen University, Shenzhen Second People's Hospital, Shenzhen, China; ^2^Department of Radiology and Nuclear Medicine, Erasmus Medical Center, Rotterdam, Netherlands; ^3^Department of Epidemiology, Erasmus MC University Medical Center, Rotterdam, Netherlands; ^4^Department of Medicine and Therapeutics, Chinese University of Hong Kong, Hong Kong, China; ^5^Department of Imaging and Interventional Radiology, Chinese University of Hong Kong, Hong Kong, China; ^6^Department of Health Technology and Informatics, The Hong Kong Polytechnic University, Hung Hom, Hong Kong

**Keywords:** intracranial arterial calcification, stroke recurrence, stroke, atherosclerosis, Agatston score, post-stroke mortality

## Abstract

**Background:** Intracranial arterial calcification (IAC) is highly prevalent in ischemic stroke patients. However, data on the association of IAC with stroke recurrence and mortality remains limited. We examined the effect of IAC on the long-term recurrence of stroke and the risk of post-stroke mortality.

**Methods:** Using a prospective stroke registry, we recruited 694 patients (mean age 71.6 ± 12.4; male sex 50.3%) since December 2004. IAC was visualized using the computed tomography exam that was made at hospital admission and was quantified with the Agatston method. All patients were regularly followed up till July 2016. The impacts of IAC on stroke recurrence and mortality were assessed using Cox-regression models with adjustments for age, sex, and relevant cardiovascular risk factors.

**Results:** During a median follow-up period of 8.8 years, 156 patients (22.5%) suffered a recurrent stroke and 84 died (12.1%). We found that a higher IAC Agatston score related to a higher risk of stroke recurrence (HR per 1-SD increase in IAC: 1.30; 95% CI, 1.08–1.56, *p* = 0.005) and a higher risk of post-stroke mortality (HR per 1-SD increase, 1.44; 95% CI, 1.06–1.96, *p* = 0.019). After investigating etiology-specific risks of stroke-recurrence, we found that a higher IAC Agatston score specifically associated with small-vessel occlusive stroke.

**Conclusions:** IAC is a strong risk factor for recurrent stroke and post-stroke mortality. Among stroke subtypes, IAC relates to higher risk of stroke recurrence among patients with small-vessel disease, which indicates chronic calcification detected in large cerebral arteries may have potential effects on the cerebrovascular beds extending to small vessels.

Worldwide, stroke is one of the leading causes of death (1), and permanent disability (2). Ischemic stroke has a complex etiology with many contributing causes such as large-artery atherosclerosis or small-vessel occlusion or cardiac emboli. Effective prevention could significantly reduce the rates of stroke recurrence and mortality (3), which may benefit from the validation of an useful imaging biomarker.

Intracranial arterial calcification (IAC) is frequently observed on non-contrast computed tomography (CT), which is a routine imaging investigation for every patient with suspicious stroke. Our previous clinical studies found a nearly 70% prevalence of cerebral arteries calcification in Chinese population (4) and a prevalence of over 80% among stroke patients (5). Inconsistent with its high prevalence, the effects of IAC on ischemic stroke has not received much attention. A recent population-based study in White individuals demonstrated the role of IAC in increasing the risk of a first stroke (6). Assessing IAC by using quantitative Agatston score, our recent study found that IAC was closely associated with cerebral artery stenosis and intracranial micro-embolism (7–9). However, data on its effects on stroke recurrence and mortality after stroke remain scarce.

Considering that IAC is a chronic disease within cerebrovascular system, this prospective study based on a local stroke registry aimed to investigate the effects of IAC on stroke recurrence and vascular mortality caused by ischemic stroke.

## Methods

### Setting

All consecutive patients that were admitted to the Prince of Wales Hospital between 1 December 2004 and 31 March 2005 with ischemic stroke or transient ischemic attack (TIA) were included in the current study. This study was part of the Prince of Wales Hospital stroke registry which was described in detail elsewhere (10). In brief, the Prince of Wales Hospital was the principal teaching hospital of the Chinese University of Hong Kong and was a tertiary referral center for the New Territories of Hong Kong. The acute stroke unit (ASU), to which all patients with stroke or TIA in this study were admitted, provided all the stroke beds available in the region (population size: 650,000). This study provided a recruitment method that was almost identical to that of a population study.

For the current study, we excluded patients whose incident stroke was due to venous infarcts, intracranial hemorrhage, subdural hematoma or subarachnoid hemorrhage. Patients with other potential causes of IAC, such as hyperparathyroidism or end-stage renal failure were excluded. Patients who refused secondary prevention with antiplatelet or anticoagulant therapies were excluded. Thirty patients were excluded due to obvious artifacts in the CT images that were not measurable for IAC. Four patients were excluded due to emigration outside the geographical area and could not be reached. This left 694 patients in the current study.

This study was approved by the institutional review board (the Clinical Research Ethics Committee of the Chinese University of Hong Kong).

### Assessment of Intracranial Arterial Calcification

At admission, all patients underwent a non-contrast CT examination of the skull, which was used to assess IAC. All examinations were performed on a 16-slice multi-detector row CT system (Light speed 16 plus, General Electric, Milwaukee, WI, USA) with the following scan parameters: 140 kVp, 170 mAs, 2 s per rotation. Axial images were reconstructed at 0.625 mm intervals.

We evaluated IAC Agatston score using a semi-automatic procedure which we previously described (7, 8). In brief, CT source images for each patient were reconstructed to three dimensional (3D) images by MATLAB (R2015a, the MathWorks Corporation, MA, USA), and subsequently transferred to 3D software (Analyze, version 12.0, AnalyzeDirect Inc., KS, USA) for IAC segmentation with “seeding method” and manual editing. Then IAC volume was automatically generated by ITK-SNAP (version 3.4.0, open source software, www.itk-snap.org) (11). Next, 3D CT images with IAC segments were reconstructed at 3 mm intervals by MATLAB. Finally, IAC Agatston score was automatically generated using a custom-made program with the Agatston algorithm (12). According to this algorithm, on each 3 mm-thick CT slice, a weighted value was assigned to the highest artery calcium density within the slice. Weighted value of 1 for 130–199 Hounsfield units (HU), 2 for 200–299 HU, 3 for 300–399 HU, and 4 for 400 HU or greater. This weighted value was then multiplied by the area of calcification in the same slice, and total Agatston score is a summed result of all CT slices.

We defined IAC as hyperdense foci with attenuation number ≥130 HU within any of the following cerebral arteries: right and left intracranial internal carotid artery (IICA), right and left anterior cerebral arteries (ACA), right and left middle cerebral arteries (MCA), right and left posterior cerebral arteries (PCA), right and left vertebral arteries and basilar artery. The results were evaluated by an experienced neurologist blinded to all the clinical data of the study population.

### Follow-Up of Stroke Recurrence and Post-stroke Mortality

Patients admitted to the ASU were followed up using Clinical Management System (CMS), a territory-wide and networked clinical records system which was utilized by the Hospital Authority throughout all government hospitals in Hong Kong. All study patients who were discharged from the hospital were followed up regularly till June 1, 2016 for ischemic stroke recurrence and post-stroke mortality. Deaths classified as due to stroke causes were death from stroke that occurred within 7 days after the current stroke or another stroke during follow-up period. A search of the patient's serial CMS records was performed to identify any development of recurrent ischemic stroke or related mortality across the Hong Kong government hospital system.

### Clinical Data and Stroke Subtypes

Clinical data of demographic characteristics, stroke risk factors, and stroke etiology of the patients were retrieved from the data-collection sheets of stroke registry collected on entry and obtained by record linkage to the CMS. Demographic characteristics included age and sex. Stroke risk factors for each patient were documented regarding the following: hypertension, diabetes mellitus, hyperlipidemia, atrial fibrillation, ischemic heart disease, two or more ischemic stroke history and ever smoking. The stroke etiology (TOAST, Trial of ORG 10172 in Acute Stroke Treatment criteria) (13) was classified as large-artery atherosclerosis, small-vessel occlusion, cardioembolism, other determined etiology, undetermined etiology, and two or more causes identified.

### Statistical Analysis

Given the skewed distribution of the IAC Agatston score we applied a natural log-transformation after 1 was added to the non-transformed values to deal with zero calcium scores [Ln (IAC + 1.0)]. Cox proportional hazards regression models were used to investigate the association of the presence of IAC and IAC Agatston scores with stroke recurrence and post-stroke mortality. In the first model we show the crude, unadjusted effect estimates. In the second model we adjusted all analyses for age, sex, hypertension, diabetes mellitus, atrial fibrillation, hyperlipidaemia, ischemic heart diseases, two or more ischemic stroke history and ever smoking. Next, we analyzed the association of IAC Agatston score with stroke subtypes (large-artery atherosclerosis, small-vessel occlusion, cardioembolism). Finally, we used Kaplan-Meier curves and log-rank tests for estimation of stroke recurrence and post-stroke mortality for patient groups with higher (>=median score) or lower (< median score) IAC Agatston scores. A significance level of 0.05 was used for all analyses. The SPSS software SPSS (version 16.0, IBM SPSS Statistics, Chicago, U.S.A.) was employed for this study.

## Results

The baseline characteristics of the study population are provided in Table 1. The mean age of the patients was 72 years (age range: 28–101 years) and 49.7% were females. The most prevalent stroke-etiology was large-artery atherosclerosis (40.0%, 233/582). Small-vessel occlusion was found in 167 patients (28.7%), cardioembolic stroke in 124 patients (21.3%), other determined etiology in 10 patients (1.7%), and cryptogenic causes in 5 patients (0.9%). Two or more causes were identified in 43 patients (7.4%). IAC was found in 643 patients (92.7%) (Table 2).

**Table 1:** Baseline characteristics.

**Characteristic**	**Total (*n* = 694)**
Vascular risk factors	
Age (years) (mean/SD)	71.6 (12.4)
Sex (male), %	349 (50.3)
Hypertension, %	503 (72.5)
Diabetes mellitus, %	232 (33.4)
Hyperlipidaemia, %	360 (51.9)
Atrial fibrillation, %	148 (21.3)
Ischemic heart diseases, %	98 (14.1)
Two or more ischemic stroke history, %	170 (24.5)
Ever smoking, %	195 (28.1)
TIA	112 (16.1%)
Ischemic stroke	582 (83.9%)
TOAST classification	
Large-artery atherosclerosis	233 (40.0%)
Small-vessel occlusion	167 (28.7%)
Cardioembolism	124 (21.3%)
Other determined etiology	10 (1.7%)
Undetermined etiology	5 (0.9%)
Two or more causes identified	43 (7.4%)
IAC characteristics	
Presence of IAC, %	643 (92.7)
IAC volume (mm^3^) (median/IQR)	169.3 (40.9–431.7)
IAC Agatston score (median/IQR)	64.8 (14.8–180.7)

*IAC, intracranial arterial calcification; SD, standard deviation; IQR, interquartile range*.

**Table 2:** Prevalence of intracranial artery calcification in 694 patients.

**IICA**	**ACA**	**MCA**	**PCA**	**Basilar artery**	**Vertebral artery**	**Any intracranial arteries**
641 (92.4%)	1 (0.1%)	25 (3.6%)	0 (0%)	20 (2.9%)	272 (39.2%)	643 (92.7%)

### Stroke Recurrence and Post-stroke Mortality

During a median follow-up period of 8.8 years (interquartile range 3.2–11.1 years), 156 patients (22.5%) suffered a recurrent stroke and 84 (12.1%) died within 7 days after the initial stroke.

Table 3 shows the associations between IAC characteristics and risks of stroke recurrence. We found that IAC Agatston score was related to a higher risk of recurrent stroke independent of cardiovascular risk factors (HR per 1-SD increase in IAC, 1.30; 95% CI, 1.08–1.56, *p* = 0.005) (Table 3, model 2).

**Table 3:** Association of IAC with stroke recurrence and mortality after stroke.

	**Ischemic stroke (*****n*** **=** **156)**		**Stroke death (*****n*** **=** **84)**	
**IAC characteristic**	**HR (95% CI)**	***P*-value**	**HR (95% CI)**	***P-*value**
Presence of IAC				
Model 1	1.80 (0.88, 3.66)	0.107	7.49 (1.04, 53.83)	0.045
Model 2	1.23 (0.57, 2.66)	0.599	3.17 (0.42, 23.79)	0.262
IAC Agatston score, per				
1-SD increment				
Model 1	1.40 (1.17, 1.67)	0.000	1.756 (1.34, 2.30)	0.000
Model 2	1.30 (1.08, 1.56)	0.005	1.441 (1.06, 1.96)	0.019

*Model 1: the crude, unadjusted effect estimates. Model 2: adjusted for age, sex, hypertension, diabetes mellitus, atrial fibrillation, hyperlipidaemia, ischemic heart diseases, two or more ischemic stroke history and ever smoking*.

*IAC, intracranial arterial calcification; HR, hazard ratio; CI, confident interval*.

For the associations between IAC characteristics and risks of post-stroke mortality, we found that IAC Agatston score was independently related to a higher risk of post-stroke mortality (HR per 1-SD increase in IAC, 1.44; 95% CI, 1.06–1.96) (Table 3, model 2).

### Stroke Recurrence in Patients With Stroke of Different Etiological Subtypes or TIA

We found 133 (22.9%) recurrence stroke in 582 stroke patients, and 23 (20.5%) in 112 TIA patients (Table 4). Among the 133 recurrence cases, 54 (23.2%) were found in patient with index large-artery atherosclerotic stroke, 34 (20.4%) in patient with index small-vessel occlusive stroke, and 27 (21.8%) with index cardioembolic stroke.

**Table 4:** Cox proportional hazard model for predictive value of IAC Agatston score on patient groups with index TIA or different major stroke subtypes.

**Category**	**NO**.	**Re-stroke during follow-up period**	**Crude HR (per 1-SD increase in IAC)**	***P*-value**	**Adjusted HR (per 1-SD increase in IAC)**	***P*-value**
All patients	694	156 (22.5%)	1.40 (1.17, 1.67)	0.000	1.36 (1.10, 1.67)	0.004
TIA	112	23 (20.5%)	1.27 (0.85, 1.89)	0.237	1.20 (0.76, 1.91)	0.435
Ischemic stroke	582	133 (22.9%)	1.41 (1.16, 1.72)	0.000	1.39 (1.09, 1.76)	0.007
Stroke subtype	582					
Large-artery atherosclerosis	233	54 (23.2%)	1.30 (0.94, 1.78)	0.108	1.20 (0.83, 1.72)	0.328
Small-vessel occlusion	167	34 (20.4%)	1.48 (1.02, 2.16)	0.040	1.67 (1.06, 2.64)	0.027
Cardioembolism	124	27 (21.8%)	1.57 (0.92, 2.70)	0.099	1.06 (0.58, 1.95)	0.842

*HR, Hazard ratio*.

*Adjusted for age and hypertension*.

For patients with index small-vessel occlusive stroke, IAC Agatston score was found to be associated with a higher risk of stroke recurrence (HR per 1-SD increase in IAC, 1.67; 95% CI, 1.06–2.64, *p* = 0.027) independent of age and hypertension.

### The Kaplan–Meier Risk of Stroke Recurrence and Post-stroke Mortality

**Figure 1:**
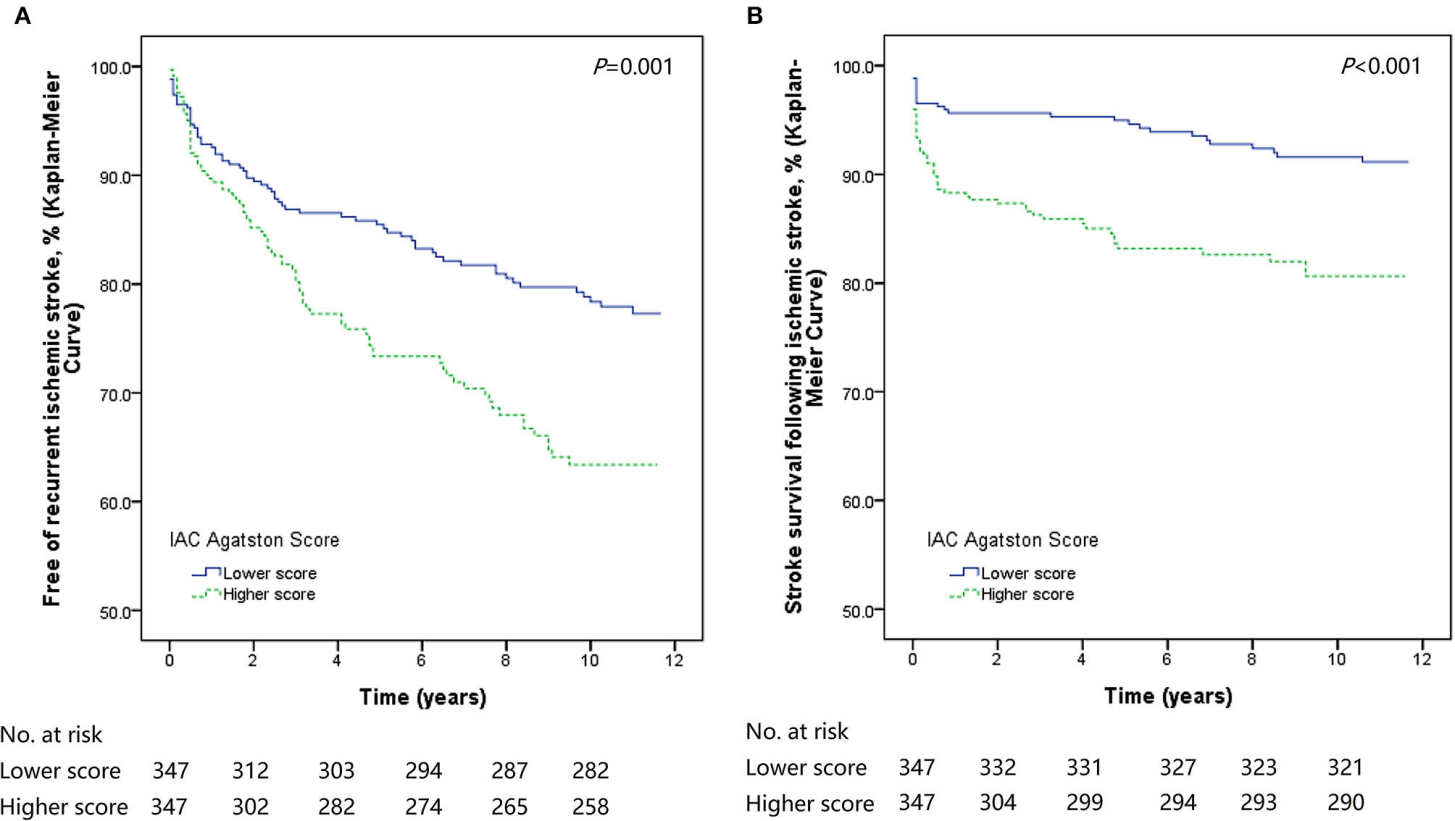
Kaplan-Meier survival plots of stroke recurrence and stroke death. Patients with higher IAC Agatston score had significantly poorer outcomes (recurrent stroke or stroke death) than those with lower score. **(A)** Kaplan-Meier survival plot for patients free of recurrent stroke. **(B)** Kaplan-Meier survival plot for stroke survival.

The Kaplan-Meier curves and log-rank tests showed significantly higher rates of stroke recurrence (*P* = 0.001) and lower rates of post-stroke survival (*P* < 0.001) in patients with higher IAC Agatston score (Figure 1). In this group, the risk of developing recurrent stroke was 9.2% at 1 year, 21.0% at 5 years, and 25.6% at 10 years. While for patients with lower IAC Agatston score, the risk was much lower, with 6.9% at 1 year, 13.5% at 5 years, and 18.7% at 10 years.

## Discussion

In this cohort study, we found IAC was associated with the risk of recurrent stroke and post-stroke mortality during over 10 years of follow-up. In addition, index small-vessel occlusive stroke patients who had higher IAC score were particularly vulnerable for another stroke.

In the term of IAC impact on ischemic event risk, Mak et al. (5) in a prospective study involving 60 Chinese stroke patients failed to verify the association between IAC and stroke recurrence (14). Different from the study involving Asians, a French research group in 2011 followed up 302 stroke patients over 1 year and demonstrated that a visual IAC score might constitute a risk factor of total future major clinical events (15). Similarly, Ovesen et al. (16) found that the severity of IAC (graded as the number of calcified cerebral arteries) predicted an increased risk of total recurrent ischemic events in a Danish population. However, in these early studies the direct association between IAC and ischemic stroke was not shown. One explanation might be that, IAC was qualitatively assessed with visual scales in these studies, but not quantified, which failed to fully reflect the IAC characteristics. Recently with a quantified software, the Rotterdam Study–a large population-based cohort study in a general community—established the IICA calcification volume to be a major risk factor for a first stroke in a White population (6). Symptomatic intracranial atherosclerotic stenoses patients have recently been found to experience more cerebrovascular events, event under best prevention management, compared to asymptomatic ones with similar vascular risk factors (17). In this study, we concerned about the stroke population who had a higher incidence of IAC. With a quantitative-evaluated IAC Agatston score reflecting both calcium volume and density information, our study demonstrated the association between IAC and recurrent ischemic stroke. The important values of IAC Agatston score may help high risk patient's stratification and management optimization in clinical practice.

In the field about prognostic value of IAC on specific stroke subtypes, little was known. A cross-sectional subgroup analysis based on the Rotterdam Study showed that compared to lacunar ischemic strokes (small vessel disease), non-lacunar strokes is associated with a larger aortic arch calcification volume (18), which confirmed extent arterial calcifications differ on different etiologies. Different to this study, we longitudinally followed up the stroke patients. Our study found that among different stroke etiological subtypes, IAC was independently related to stroke recurrence in patients with index small-vessel occlusive stroke. This finding implicated that IAC play various roles on different causes of stroke and small-vessel occlusive stroke patients with more severe IAC may be particularly vulnerable for another stroke.

For arterial calcification detected on CT, there are now three recognized patterns: intimal calcification which is indicative of a proxy for intracranial atherosclerosis documented in our previous histological study, medial calcification described by Mönckeberg, and internal elastic lamina calcification which is associated with stiffening of the arterial wall and increased pulse pressure (19–23). This study quantitatively evaluated IAC on CT, which include all there above patterns. Although the latter two patterns are currently considered non-atherosclerotic calcification, they could contribute to increased mechanical stress on atherosclerotic plaques, and effect the plaque sequelae potentially. This may be one explanation for the association between quantitatively-evaluated IAC and subsequent events. Besides, association between extent of arterial calcification and stroke prognosis in different ethnicities would also be an interesting point worthy of future attention.

This study presents some strengths. First, consecutive patients are studied on a prospective registry. Second, we use quantitative methods to evaluate arterial calcification, which contain both calcium volume and density information, and reflect the severity of IAC more objectively and authentically than visual scales. Third, we evaluate IAC on non-contrast head CT, which is non-invasive and easily acquired in general hospitals. Finally, this is a very long-term study and the majority of patients are successfully followed up. On the other hand, some limitations exist in this study, including overestimation of extent of vascular calcification in CT imaging, the absence of stroke severity on admission, the absence of treatment for stroke, monocentric study, and the small sample size especially among different stroke subtypes. Yet, as a preliminary study it can provide considerable value for reference in future clinical research.

## Conclusion

The quantitatively-evaluated IAC relates to long-term stroke recurrence and vascular mortality. Among stroke subtypes, IAC is associated with recurrent stroke among small-vessel occlusive patients, which indicates choric calcification detected in large cerebral arteries may have potential effects on the cerebrovascular beds extending to small vessels.

## Data Availability Statement

The original contributions presented in the study are included in the article/supplementary material, further inquiries can be directed to the corresponding author.

## Ethics Statement

The studies involving human participants were reviewed and approved by the Clinical Research Ethics Committee of the Chinese University of Hong Kong. Written informed consent for participation was not required for this study in accordance with the national legislation and the institutional requirements.

## Author Contributions

XW: conceived and designed the study, collection of data, imaging analysis, data analysis and interpretation, and manuscript writing. XC: conception and design, data interpretation, reviewed and edited the manuscript, and final approval of manuscript. DB: data interpretation, reviewed, and edited the manuscript. LR: data interpretation. TL: collection of clinical data. WC and JA: imaging analysis. LW: conception and design and financial support. All authors: read and approved the manuscript.

## Conflict of Interest

The authors declare that the research was conducted in the absence of any commercial or financial relationships that could be construed as a potential conflict of interest.
